# Solid waste composition and its management: A case study of Kirtipur Municipality-10

**DOI:** 10.1016/j.heliyon.2023.e21360

**Published:** 2023-11-02

**Authors:** Prakash Awasthi, Gopi Chataut, Ram Khatri

**Affiliations:** a Institute of Agriculture and Animal Science, Tribhuvan University, Nepal; b School of Environmental Science and Management, Nepal

**Keywords:** Composition, Management, Municipality, Solid waste

## Abstract

The world population is expanding in line with the general trend. The demand for goods is currently higher than it has ever been before. This has resulted in the production of more waste than ever before. The problem of waste management is not new for humans but the complexity of the issue has increased more in present days. The research was focused on composition of solid waste and its management in Kirtipur-10. The research was conducted in ward 10 of Kirtipur municipality to know the ground reality of solid waste in households. The research aimed to understand the solid waste situation, its composition, problems and finding possible solutions to the problems. Household survey, Focused group discussion were conducted during November month of 2022 to collect data. 75 household were taken as sample for research. Wastes, collected from all of the groups of households, were segregated and weighed. Household solid waste (HSW) was comprised of seven categories of waste with organic waste being the largest component (44 %), plastic (13 %), paper (11 %), Glass (10 %), rubber (9 %), others (7 %) and Hazardous waste (6 %). Municipal authorities are usually the responsible agencies for solid waste collection and disposal, but the magnitude of the problem is well beyond the ability of any municipal government to tackle. There is strong dissatisfaction among municipal resident on waste management by local government. Looking at the composition of waste produced, composting of waste at home level can bring a sustainable solution to the problem. The solid waste management system in Kirtipur has low efficiency and needs to be improved.

## 1 Introduction

Population expansion has contributed to a large increase in the generation of municipal solid waste (MSW), which is connected with severe environmental and public health problems around the world [1]. The amount of MSW generated is affected by a variety of factors, including food preferences, standard of living, level of commercial activity, and season [2]. Any unwanted or excluded materials generated by various human activities are considered solid waste. It can be classified based on its composition (organic material, glass, metal, plastic, or paper) or its hazard potential (toxic, non-toxic, flammable, radioactive, or infectious). Solid waste is a byproduct of human activity that is expected to increase as the global population grows, urbanization accelerates, living standards improve, and consumption patterns shift. In recent decades, solid waste management (SWM) has become an alarming concern for land degradation, biodiversity loss, air pollution, sanitation, and the spread of infectious diseases in many developing country cities [3].

The management of municipal solid waste (MSWM) includes all of the following activities: collection, transfer, resource recovery, recycling, and treatment. Protecting public health, enhancing environmental quality, fostering sustainability, and boosting economic output are the main goals of MSWM [4]. Public health and environmental management receive very little funding from the national budget. For more than a decade, Nepal's media, government, and non-governmental groups have been debating the hot problem of solid waste management, particularly the search for suitable sites for the land filling of the MSW produced by the municipalities inside the valley [5]. The seriousness of waste management is highly needed inside Kathmandu which occupies 10 of the developing municipalities of the country and has high waste production rate. In the past, solid waste was not a major issue in Kathmandu Valley. People in Kathmandu Valley had their own system for managing household waste, which included the circulation of organic waste between cities and rural areas nearby. As a result of the valley's growing population, changing lifestyles, and consumer habits, SWM has become one of the valley's key environmental challenges [6]. Hence, the primary issues to be addressed in Kathmandu Metropolitan City are proper municipal solid waste management (MSWM) systems and innovative renewable energy alternatives (Kathmandu Metropolitan City). The current MSWM system is limited to waste collection and disposal in landfills, which has long had a negative impact on the environment and public health [7].

Over time, there has been a continuous rise in the number of people living in urban areas all over the world [8]. In 2018, the world's urban population was 55 % of total population, but a research from the Department of Economic and Social Affairs of the United Nations has predicted that number will rise to 68 % by 2050 [9]. As per the reports more than 90 % of growth will occur in Asia and Africa. The increase in urban population, combined with economic expansion and increased living conditions, has resulted in massive amounts of waste being generated in developing-country cities [10]. Increasing population levels, a booming economy, fast urbanization, and an increase in community living standards have all contributed to a significant increase in the rate of municipal solid waste output in developing countries [10]. Migration of people from rural areas to urban areas has resulted in unplanned settlement. Due to negligence of local authorities on environmental impact from Municipal Solid Waste (MSW) pollution level has increased tremendously deteriorating land, water and air. On the other side, MSW management processes such as composting, burning, and landfills emit greenhouse gases into the environment such as methane, nitrous oxide, and carbon dioxide [11].

The problem of solid waste in Kirtipur is similar to that of urbanizing towns in the world. Even though Kirtipur municipality has a public-private partnership (PPP) system for trash management, the oligopoly of commercial waste collectors has deprived some communities from waste collection service [12]. The optimization of MSW disposal service is hindered by inadequate funding, deteriorating infrastructure, and poorly maintained MSW collecting trucks. Municipalities, who are often in charge of waste management in cities, face the difficulty of providing residents with an effective and efficient system. They frequently face challenges that are beyond the municipal authority to address [13]. The average MSW collection efficiency is 62 %, whereas the disposal rate is only 37 % in Nepal [14].

Solid waste management is a burning issue in developing and rapidly urbanizing cities in the world. In case of Nepal also big cities like Kathmandu, Pokhara, Kirtipur are facing similar problems. The solutions to these problems associated with waste can only be solved through detailed research on the waste composition and then its management. This research will bring the ground data of composition of solid waste and ways it is being managed. This way the loop holes can be highlighted and solved. The problem of Kirtipur municipality is more specific as it is crowded with students from all over Nepal and has complex demographic characters. This research will help to improve the condition of households through efficient trash management. It would also assist the relevant agencies in reforming and improving waste management policies. This can also help to improve socioeconomic position.

## 2 Literature review

### 2.1 General information

Waste includes all items that people or businesses no longer need and intend to get rid of or have already discarded.

Solid waste also includes garbage produced by residential, commercial, industrial, or institutional building, as well as demolition and municipal services. However, this definition varies widely amongst waste studies, and some sources, such as industrial, construction and demolition, as well as commercial and municipal services, are frequently excluded [15].

MSW is defined by engineers as materials derived from residential and commercial sources [16]or things that have lost their value to the holder [17]. According to anthropologists, waste is real evidence of a society, that "What people have owned and thrown away can speak more eloquently, informatively, and truthfully about the lives they lead than they themselves ever may" [18]. According to ecologists, there is no waste in nature [19], and industrial ecologists consider waste to be "a right thing in a wrong place, like a pig in the parlor instead of the barnyard" [20]. The treatment of waste reflects its description; refuse workers carrying rubbish to a landfill treat it as worthless, whereas waste pickers recovering materials from refuse treat it as ore [21]. Regardless of the various definitions of trash, its existence and prevalence are undeniable.

### 2.2 Solid waste management scenario in global context

According to the different literatures, solid waste management is a big concern in both developed and developing countries. The world is urbanizing at an unprecedented rate. These changes present a challenge to cities, which are responsible for waste management in a socially and environmentally responsible manner. Local waste characteristics, which vary with cultural, meteorological, and socioeconomic elements, as well as institutional capabilities, are critical to effective waste management solutions. Waste management is becoming more regionalized and structured on a global scale. Waste is typically controlled formally at the municipal or regional level in developed nations, where residents produce significantly more waste than other citizens. In less-industrialized countries, where citizens generate less trash, which is largely biogenic, waste is managed by a mix of formal and informal players [22]. There were 2.9 billion urban residents ten years ago who created around 0.64 kg of MSW per person each day (0.68 billion tonnes per year). According to the research, these volumes have already climbed to almost 3 billion inhabitants, generating 1.2 kg per person every day (1.3 billion tonnes per year). By 2025, there will most certainly be 4.3 billion urban dwellers creating approximately 1.42 kg/capita/day of municipal solid trash [23].

Despite a history of environmental disasters that have affected Americans, landfills remain the most practical and cost-effective method of rubbish disposal. Some developed countries, such as Japan and Canada, are inevitably shifting their waste disposal strategies away from landfills and toward incineration [24].

A case study in one of the emerging economy in world in India was done which showed in 2015, urban India generated approximately 62 Mt of solid trash (450 g per capita per day). A total of 82 % of MSW was collected, with the remaining 18 % being trash. Only 28 % of the waste collected was processed, with the remaining 72 % deposited publicly. Waste collection efficiency in big metropolitan centers ranges between 70 % and 95 %, whereas it is less than 50 % in numerous smaller communities [25].

Comparative analysis showed that the organic component is more than 65 % of total waste in less developed countries such as Bangladesh or Pakistan, whereas it is less than 30 % in emerging countries such as Japan and the Republic of Korea [26]. According to research, increased organic percentages tend to increase the density and moisture content of waste streams, which are significant factors to consider when developing collection, transportation, and treatment systems. The moisture content of garbage is believed to be 20–30 % in developed countries, but 50 % or greater in developing ones [27]. Another comparative analysis done between Europe, USA and Asia revealed that Europe had the best municipal solid waste management (MSWM) system, whereas the US had a remarkably close but lower score. Asia received the lowest score, which was insignificant in comparison to the other two continents [28].

### 2.3 Solid waste management in Asian region

Asia consists of both developed and developing countries. Asia's developing countries have an annual urban population growth rate of about 4 %. The difficulties created by municipal solid waste are particularly complex in Asia since most nations are quickly urbanizing, with population increases ranging from 30 to 50 % [29]. The Asia Pacific area has 4.1 billion people, accounting for more than half of the world's population of 7.5 billion [30]. Increased urban population growth leads to increased food consumption, economic development, urbanization, and industrialization, all of which are major contributors to increased municipal solid waste (MSW) output in Asian countries [15]. Asian countries generate roughly 4.4 × 109 t/y of solid waste per year and spend around 25 M US$ per year on MSW management (MSWM) [23,31]. Plastic waste in municipal solid waste and industrial solid waste was predicted to reach 79 Mt and 42 Mt in Asia, respectively [32]. It is also observed that the amount of waste produced strongly reflects the people's economic standing. Asia is such a huge and diverse region that it escapes categorization. The data of countries producing varying waste in Asian region is shown in Table 1 [33].

**Table 1:** Data on waste generated based on income with characterized compositions of some Asian countries [28].

Country	GDP(PPP) per capita estimated for 2007 (USD)	Waste generation (Kg/capita/day)	Composition (% wet weight basis)						
			Biodegradable	Paper	Plastic	Glass	Metal	Textile	Inert and others
Hongkong	37,385	2.25	38	26	19	3	2	3	9
Japan	33,010	1.1	26	46	9	7	8		12
Singapore	31,165	1.1	44.4	28.3	11.8	4.1	4.8		6.6
Taiwan	31,040	0.667	31	26	22	7	4	9	
South Korea	23,331	1	25	26	7	4	9	29	
Malaysia	12,702	0.5–0.8	40	15	15	4	3	3	20
Thailand	9426	1.1	48.6	14.6	13.9	5.1	3.6		14.2
China	8854	0.8	35.8	3.7	3.8	2	0.3		47.5
Philippines	5409	0.3–0.7	41.6	19.5	13.8	2.5	4.8		17.9
Indonesia	5096	0.8–1	74	10	8	2	2	2	2
Srilanka	5047	0.2–0.9	76.4	10.6	5.7	1.3	1.3		4.7
India	3794	0.3–0.6	42	6	4	2	2	4	40
Vietnam	3502	0.55	58	4	5.6	1.6	1.5	1.8	27.5
Lao PDR	2260	0.7	54.3	3.3	7.8	8.5	3.8		22.5
Nepal	1760	0.2–0.5	80	7	2.5	3	0.5		7

A case study in China showed that MSW composition is complicated with a moisture level of more than 50 % and is dominated by 52.8–65.3 % kitchen trash, 3.5–11.9 % paper, and 9.9–19.1 % rubber and plastics. The MSW management system in China needs to be enhanced; MSW is processed by 52 % landfill, 45 % incineration, and 3 % composting methods, with utilization efficiency in China significantly lower than in industrialized nations [34]. The total volume of MSW surged from 31.3 million tonnes in 1980 to 212 million tonnes in 2006, and the waste generation rate increased from 0.50 kg per population per day in 1980 to 0.98 kg per capita per year in 2006 [35].

### 2.4 Solid waste management practices and scenario in Nepal

A Study on Solid Waste Composition in Kanchanpur District showed a total of 446.7 kg of solid garbage was collected, with organic waste accounting for the lion's share (66.4 %). Households had the highest daily generation of overall solid trash (23.3 %), followed by hotels and lodges (20.4 %). The commercial centers generated the most garbage of all categories. The practice of dumping crude waste in open and deserted locations has been identified as a concern to human health, generating ecological imbalances and polluting the land, water, and air [36].

A case study in Tulsipur showed solid waste management has been primitive and unorganized. For 11 years, the city implemented direct waste discharge on a riverbank, followed by present waste disposal in a creek with no soil cover or leachate treatment. Using cluster sampling approaches, over 100 Tulsipur Municipality houses were researched in order to describe household solid waste and survey waste management practices. According to the waste composition study, 46 % of home solid waste is organic waste, 11 % is dirt and building debris, 10 % is plastics, 7 % is glass, 6 % is paper and paper products, 5 % is metals, and 5 % is rubber and leather. The remainder was made up of textiles (1 %), hazardous trash (1 %), and other waste (8 %). Tulsipur creates 330.4 g of household solid trash per day per person. The waste composition indicates that, while organic wastes remain the majority, recyclable products have appeared in recent years [37].

A case study done in Pokhara showed per capita trash generation rate was found ranging from a low of 0.016 kg to a high of 0.082 kg, with an average of 0.035 kg. The waste composition research revealed that landfill matter accounted for the largest fraction, accounting for 39.23 % of landfill waste, 35.14 % of reusable/recycle waste, and 25.63 % of organic waste. The challenges of solid waste management include an increase in waste generation as well as per capita generation in relation to population growth and catchment area, a scarcity of garbage collection vehicles, dead animals that are difficult to manage, a lack of proper management of medical and industrial waste and hazardous waste, and a health problem among solid waste management staff [38].

According to a survey, households in the Bharatpur Metropolitan City recognized garbage collection frequency, scheduling of door-to-door waste collection services, and street cleanliness as important municipal waste collection elements that affect their welfare and willingness to pay. Despite the fact that almost every residence (95 %) in the study area used the waste collection service, more than half (53 %) expressed dissatisfaction with the current service. Women were the key players in rubbish collection and disposal at the household level. According to the findings of the choice analysis, families prefer a defined waste collection time and waste collection bins placed at regular intervals on the streets for use by pedestrians who commonly discard garbage on the streets when bins are not accessible [39].

### 2.5 Overview of solid waste management inside valley and Kirtipur

In 1980, Kathmandu's solid waste management was reorganized, followed by a succession of foreign-aid projects. Despite international funding and multiple organizational initiatives, waste management in Kathmandu has remained in crisis.

Kathmandu's municipal solid trash generation was 0.66 kg per capita per day, or 523.8 tonnes per day, with organic garbage accounting for 71 % of household waste and hazardous waste accounting for 1 %. In comparison to peer cities, the unit cost on waste management was $2.71 capita-1 annum-1, or 1.01 % of per capita GNP. There exist waste management laws, rules, guidelines, and national and local organizations, however the laws are poorly implemented and are regularly repealed and replaced with new orders. Despite a 526.3 % overall compensation rise in 1997/98, municipal workers are the lowest paid in South Asia [40]. A case study done in 2006 showed that between 2005 and 2006, solid waste generation was 1091m3/d (245 tons/day) and 1155m3/d (260 tons/day), respectively. In 2003, the overall collection efficiency was 94 %. The majority (89 %) of Kathmandu Metropolitan City families are willing to separate their organic and non-organic waste [41].

A field survey conducted in 2003 found that the average per capita household waste generation rate in Kirtipur was 0.34 kg/person/day. This is slightly greater than Nepal's average trash generation rate, which is estimated to be 0.25 kg/person/day. According to the survey, around 95% of the garbage is organic in origin. Although it is not surprising that organic materials constitute the majority of the waste stream, 95% appears to be fairly high [42].

## 2.5 Data collection

Two types of data were collected primary and secondary. Primary data was collected through direct observation and survey while secondary from different sources. Research was focused on the separation and composition of degradable and non-degradable garbage in households. As a result, both quantitative and qualitative methods were used.

## 3 Materials and methods

The research was conducted in Kirtipur municipality of Kathmandu district. The region has been facing serious problem of waste management. Kirtipur Municipality is located in the steep section of Kathmandu District. Kirtipur is located between longitudes 27° 38′ 30″ and 27° 41′ 30” E and latitude 85° 13′ and 85° 19’ N, with elevations ranging from 1284 m to 1524 m above mean sea level. Total area of the municipality is 14.76 km^2^ [43]. The municipality now is home to total population of 65,602 (Male 55.6 % and Female 44.4 %) with average household population of 3.37. The total number of household was recorded 19,441 as per CBS records. Among all the wards, ten number ward was selected for the study where there are 3,192, households. The total population of ward 10 was found 9739.

Household were requested to collect waste for 15 days in separate plastic bag to segregate waste produced from household level. After 15 days waste was collected from household and weight separately using digital spring balance. Precaution was taken while weighing waste. There was use of face mask and disposable gloves during the process. Data obtained was manually collected in note copy and later uploaded in excel for analysis. Hand sanitizer was used to avoid contamination from any hazardous waste.

### 3.1 Primary data collection

Primary data was collected using various methods.

#### 3.1.1 Direct observation

Selected 75 households were provided with 7 plastic bags and were requested to collect and segregate waste in those buckets for 15 days. The selected wastes were categorized as: Organic waste, plastic, glass, rubber, hazards and others. This was carried from 2022 to 11-1 to 2022-11-15.

The waste collected was categorized and weighed separately using weighing machine and data was recorded.

#### 3.1.2 Household survey

Different questions were prepared based on preliminary survey and general observation of the situation of ward number 10 of Kirtipur municipality. The question mainly focused on the age of respondent, household size, waste collection, waste disposal and to know residents characteristics based on education, economic status etc.

#### 3.1.3 Focused group discussion

Focused group discussion was done to collect information on nature of problems and possible recommendations for solving the issues. It helped to get insights into people perception, satisfaction and willingness on solid waste management. Total of 6 participants representing different area and household of ward 10 of Kirtipur municipality were selected on basis of their willingness. The focused group comprised of 4 male and 2 female. FGD was composed of participant of all age group above 18 years of age who could put their opinion on the given agenda. Mixed gender and varying age group were preferred to get broad information and perspective of the participants.

## 4 Secondary data collection

Secondary data was collected using the information from Google scholar, Research Gate as well as technical reports of which authors were informed. Despite large number of articles identified for each sub-topics only limited article were selected based on relevancy and authorized data. In all about 150 articles were investigated and only 40 articles ended up being used in this research. Secondary data has been illustrated in this research with access from the author.

### 4.1 Data analysis

Both qualitative and quantitative data was gathered together from the study. Quantitative data was coded in excel sheet for the analysis and interpretation. After the data collection from altogether 75 households visited for 15 days during field survey, data was checked thoroughly and analyzed.

Average waste per kg per household per day(x) = Total waste collected from each sample household for 20 days in grams/15 days × 1000.

Kg per capita per day waste = Total sum of Average waste of each household/Total population of study Area.

Data was analyzed using excel and presented in graphical forms.

## 5 Results

### 5.1 Situation of solid waste in Kirtipur municipality

To understand the composition of solid waste in Kirtipur municipality compositional analysis was done from data collected from household level.

#### 5.1.1 Composition of solid waste generated at household level

The solid waste composition was categorized into organic waste, plastic, papers, glass, rubber, hazardous waste and other.

The data was collected from the 75 household of ward 10 of Kirtipur municipality. The duration of waste collection was 15 days. Each household were given bins to collect the waste separately.

The data clearly shows that the organic waste holds most of the waste composition which is 44 % followed by plastic 13 % and papers at third level with 11 % of total waste.

#### 5.1.2 Education status of resident

Field survey was conducted to know the education level of the resident in Kirtipur municipality. The data received from the survey has been presented in Fig. 4. The figure clearly shows that all most every respondent has received some level of formal education. 15.57 % of respondent were below SLC level of formal education. The highest portion of respondent had gained bachelor level of education which was 30.88 %. This data clearly shows that people in Kirtipur are well educated and can be hope for solid waste management.

#### 5.1.3 No of Household members

Fig. 5 clearly shows that majority of household member ranges between 4 and 7 with highest of 5 members in family which accounts for 33.57 % of total respondents. It was followed by 6 member per household which was found in 33.38 % of respondents. The minimum value was seen in 4 and 7 members per household.

#### 5.1.4 Waste collection frequency

Fig. 6 shows the frequency with which municipality and concerned authority collect waste from household. Field survey showed that major respondent were unsatisfied with municipal work as the waste collection frequency seemed uncertain as 72.5 % were respondent are in uncertain waste collection frequency category. This uncertainty from waste collection authority has made more problems in household and some household even dump their waste at river side.

The data of frequency of waste collection does not align with the time schedule provided by municipality and time agreed by waste collection companies. As per municipality, Waste collection and transportation in the ward level begins at 7:00 a.m. and continues until 11:00 a.m., varying with the private sector. Different types of vehicles owned by various private sectors collect waste from their respective locations and transport it to the dumpsite after segregation. They arrive at the transfer station between 10:30 and 11:30 a.m. Waste collection is done twice a week but the ground reality is different.

#### 5.1.5 Segregation of Degradable and non-degradable waste

Segregation of household waste is one of the important parts in proper solid waste management. When respondent were asked about if they segregate waste at household level. Most of the respondent answered with yes but the ground reality seemed different. It clearly showed that people are aware about waste segregation but due to negligence and proper policy there is no such strict system of waste segregation at household level.

### 5.2 Non-governmental sector involved in solid waste management in Kirtipur municipality

Kirtipur Waste Management and Service was founded in 2013 and specializes in the separation of recyclable waste. In 2012, the Nepalese government held a program called "Aafno gaun afai sarsafai garau" in the Kirtipur Municipality (clean your village on your own). A waste survey was undertaken in Panga, Kirtipur Municipality, which produced data, and KWMS was urged to participate in this area. The 'Polluter Pay' principle, which was first recognized as an internationally agreed principle by the Organization for Economic Cooperation and Development (OECD) in 1972, was introduced in Nepal under section 17 of the Environment Protection Act, 1997, which states that the cost of pollution and consequential costs should be borne by the person responsible for causing the pollution. The government was permitted to collect a garbage levy from persons who generate rubbish under this premise. Previously, rubbish collection was the responsibility of the municipality. Kirtipur Municipality has a dedicated account earmarked for solid waste management, and four private companies are currently in charge of solid waste management: Kirtipur Waste Management Service Pvt. Ltd., Nepal Swachchha Batabaran Sirjana Kendra, Clean Nepal Pvt. Ltd., and Swachchha Batabaran Samrakshan Samiti. The private companies provide 2 % of the collected tariff is as revenue to Kirtipur Municipality. Household waste collection and service charges range between NRs. 150 and 450 per month, depending on family size. The waste collection and services charges range from NRs. 500–4000 per month, depending on the number of students present at the schools. Similarly, charges for non-residential waste collection and service range from NRs 250–300 per month for institutions such as banks, office buildings, groceries, pharmacies, and saloons. However, service charges for the garment and carpet industries have been set at NRs. 1500–2000. The highest price for slaughterhouses has been set at NRs. 2000–3000 per month. According to the IUWM report, these four SWM entrepreneurs serve 8100 households and generated an average of 7416 tons of waste per year. Household waste, followed by commercial waste and institutional waste, was the municipality's disintegrated source of waste, with 74 % organic waste and 15 % plastic waste reported [44].

### 5.3 Focused group discussion on solid waste management

Focused group discussion was conducted in ward no 10 of Kirtipur municipality. There were total of 7 participants in the discussion session. The agenda of focused group discussion was waste management system in the ward. The objective of the discussion was.

• To bring forward the issues of solid waste management
• To understand the causes of solid waste problem
• To gain information on possible solution of solid waste problem from ground level

The response from participant showed strong disappointment from the waste management system. It was clear that resident of Kirtipur were not well trained on segregation of waste and its proper collection. The municipal vehicle carrying waste has also irregular time of waste collection of household which creates problem among resident to store waste and has been creating problem of foul smell, diseases at household level.

## 6 Discussion

Waste generation has been a problem from the time of human development and with the increase of pace of urbanization and globalization the issue has been more complex. Solid waste management is not only problem of Kirtipur or Nepal but whole planet and it is a major agenda for world to solve.

Analysis of data collected from field survey showed that the organic waste holds most of the waste composition which is 44 % followed by plastic 13 % and papers at third level with 11 % of total waste. The data clearly shows that waste comprises most of organic one and if properly segregated can be used as compost and improve land quality instead of degrading it. The composition of solid waste can help to make policies based on waste generated. The other most produced waste like plastic and papers can be recycled and reused. Comparing the obtained data with Kathmandu metropolitan shows that Household waste in Kathmandu metropolitan comprised of 51 % organic and 49 % recyclable comprising 5 % paper and paper products, 3 % glass, 1 % metal, 19 % plastic, 13 % textiles, 4 % rubber and leather and 4 % other waste in Kathmandu [45].

The study in Kirtipur has shown the average per capita per day waste of the study area to be 0.0347 kg/Person/day. This is very low compared to the Kathmandu metropolitan city which has waste generation of 0.3 kg/capita/day, with a total household waste generation of 413 tonnes/day [45].

Average waste generation per household per day was found to be0.1059 Kg and total waste generated per day was338.217 kg. The data is in line with a similar research done in 5 other municipalities of Kathmandu valley which showed Average per capita HH waste (kg day-1) of 0.15 during 2016 [46]. The data collected from this research is varying and depends upon cultural practice of household, economic level, eating and cooking habits and so on.

The result showed high level of literacy among respondents and awareness regarding the issue of waste management. The data showed that almost all the respondents are literate in kiritpur and have knowledge on waste management. Despite the fact that most of the respondents were willing to segregate waste the ground reality of waste segregation is opposite to it. Despite an average household size and normal waste generation, due to lack of proper collection and disposal the waste is becoming more of an issue.

Number of household member has direct relation with produced waste. More member means more waste production. In Kirtipur particularly, the household size ranges between 4 and 7 members, with highest comprising of 5 members in 35.57 % of total respondents. There is uncertainty in waste collection from private companies as 72.5 % respondent reported uncertain frequency of waste collection.

The data obtained from the research is in contrast with the data collected from survey by the Kirtipur municipality at house hold level.

The result conducted by Kirtipur municipality shows 77 % of organic waste composition. This is nearly double the portion from our result. Such contrast in data is due to the time of waste collection survey. Kirtipur municipality conducted the survey during rainy months of the year while this survey was done during winter month which lead to lower dry weight and it caused fluctuation in data.

## 7 Conclusion

The situation of solid waste management is very unsatisfactory and inefficient in Kirtipur. The organic waste holds most of the waste composition which is 44 % followed by plastic 13 % and papers at third level with 11 % of total waste. The most of organic waste has been produced from kitchen level. The average per capita per day waste of the study is found to be 0.0347 kg/Person/day. Average waste generation per household per day is 0.1059 Kg and total waste generated per day is 338.217 kg. All most all the resident follow similar method of waste collection which is later collected and carried away by municipal vehicle. The frequency of waste collection from municipal level is uncertain and has caused a lot of trouble to residents. The oligopoly of commercial waste collectors has caused problem to some community people as well. As an alternative to this problem, some residents prefer to utilize the organic waste in making compost at home level. The major problem in Kirtipur municipality is waste segregation and its proper policy and implementation from municipality level. The final disposal site is also not managed properly. It is generally regarded that waste management is the sole duty and responsibility of local authorities, and that the public is not expected to contribute. There are multiple stakeholder where citizens has even more important role in waste segregation for effective waste disposal.

## Data availability

**Figure 1:**
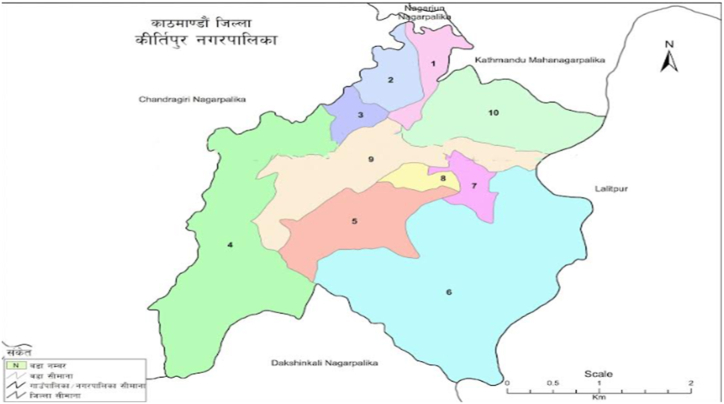
Map of study area.

**Fig. 2:**
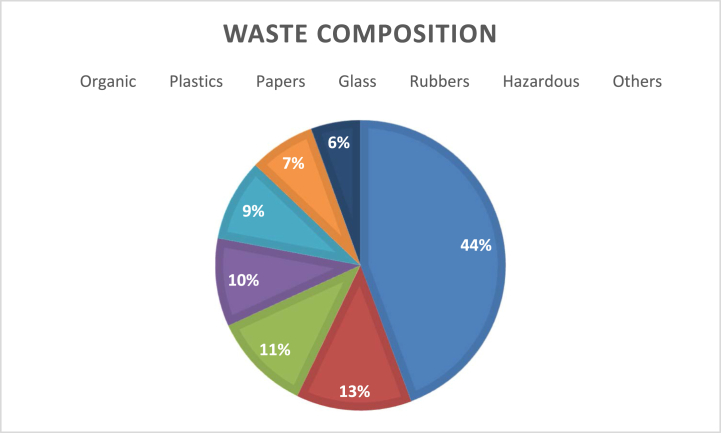
Percentage composition of solid waste at household level.

**Fig. 3:**
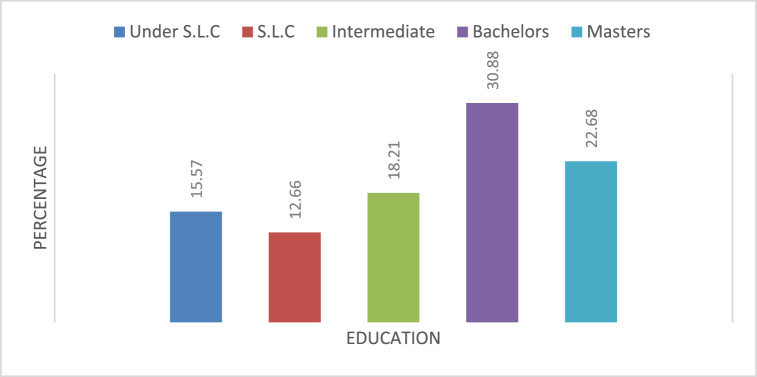
Education status of resident.

**Fig. 4:**
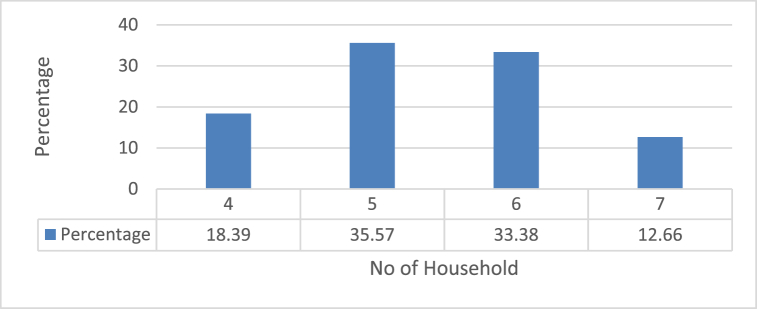
No of Household members.

**Fig. 5:**
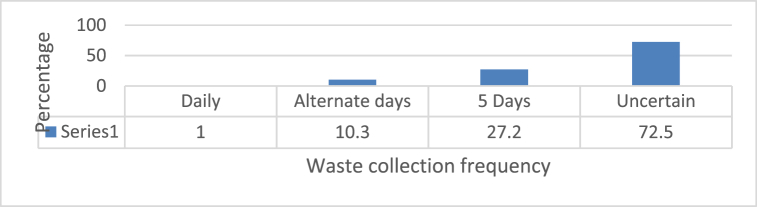
Waste collection frequency.

**Fig. 6:**
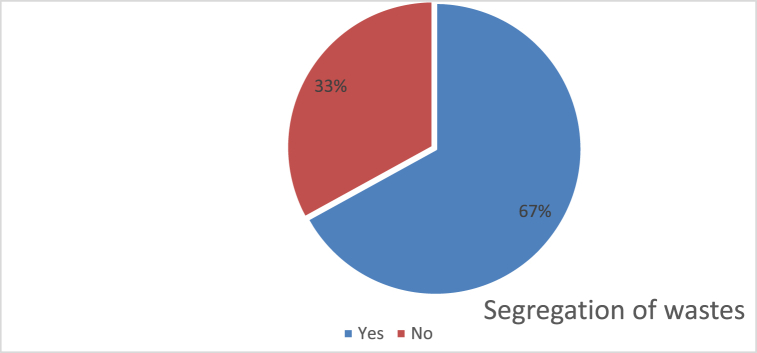
Segregation of Degradable and non-degradable waste.

**Fig. 7:**
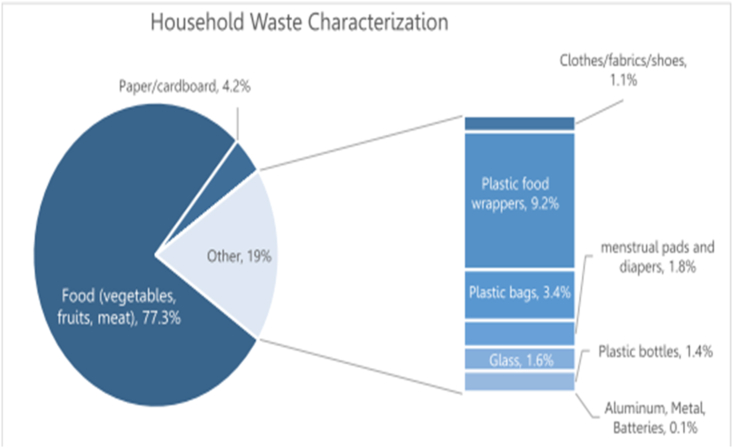
Household waste composition [46].

All the data justifying the correctness of the research are available in the manuscript. Figure 1, Fig. 2, Fig. 3, Fig. 4, Fig. 5, Fig. 6, Fig. 7 provide the experimental data and simulation results that support the study's conclusions.

## CRediT authorship contribution statement

**Prakash Awasthi:** Data curation, Project administration, Resources, Supervision, Validation, Visualization, Writing – original draft, Writing – review & editing. **Gopi Chataut:** Conceptualization. **Ram Khatri:** Resources.

## Declaration of competing interest

The authors declare the following financial interests/personal relationships which may be considered as potential competing interests:The authors declare that they have no known competing financial interests or personal relationships that could have appeared to influence the work reported in this paper If there are other authors, they declare that they have no known competing financial interests or personal relationships that could have appeared to influence the work reported in this paper.
